# Physical determinants of vault performance and their age-related differences across male junior and elite top-level gymnasts

**DOI:** 10.1371/journal.pone.0225975

**Published:** 2019-12-05

**Authors:** Christoph Schärer, Nils Haller, Wolfgang Taube, Klaus Hübner

**Affiliations:** 1 Department of Elite Sport, Swiss Federal Institute of Sport Magglingen (SFISM), Magglingen, Switzerland; 2 Department of Neurosciences and Movement Sciences, University of Fribourg, Fribourg, Switzerland; University of Pittsburgh, UNITED STATES

## Abstract

In order to perform difficult vaults in artistic gymnastics, athletes have to achieve high run-up speeds within the limited run-up distance (25m). However, the physical parameters that contribute to a high run-up speed and their age-related differences remain elusive. Hence, the aim of this study was 1) to investigate interrelations between difficulty value (D-score) and run-up kinematics of Handspring/Tsukahara and Yurchenko vaults as well as lower body power (25m-sprint, explosive and reactive strength) and 2) to explore age-related differences of these parameters across junior and elite gymnasts performing Handspring/Tsukahara vaults. For this purpose, the data (of the above mentioned parameters) of 47 top-level male elite and junior gymnasts aged 14.3 to 28.3 of performance testing, gathered over three years, were analysed. We found that D-score of Handspring/Tsukahara (n = 33) was strongly correlated with run-up speed (r = 0.79; p < 0.01). Further, 25m sprint speed (r = 0.85; p < 0.01) was significantly associated with run-up speed of Handspring/Tsukahara-vaults. There were no significant relationships with the D-score of Yurchenko (n = 14). Looking at the age-related differences of Handspring/Tsukahara, D-score increased significantly from junior to elite level (+11.6%; p < 0.01). The comparison between consecutive age-groups revealed that the U19 group had higher run-up speeds, step lengths, body weights and heights than the U17 group, while the U21 group achieved significantly higher speeds (run-up, 25m-sprint) and explosive strength than the U19 group. We concluded 1) that the optimization of important physical determinants may increase the potential to perform more difficult Handspring/Tsukahara vaults and 2) that first growth and maturation and later improvements of lower body power led to higher run-up speeds of Handspring/Tsukahara in the subsequent age-group. Therefore, based on performance testing of the lower limbs, training recommendations should be given specifically to the requirements of the competition vault.

## Introduction

Artistic gymnastics has evolved rapidly since the introduction of the unlimited scoring system in 2006 [1]. On vault for example—improvements of spring characteristics of the vaulting table and spring board, as well as continuously increasing run-up speeds, have led to a large number of very difficult vaults performed at international competitions in the last decade [2].

**Fig 1:**
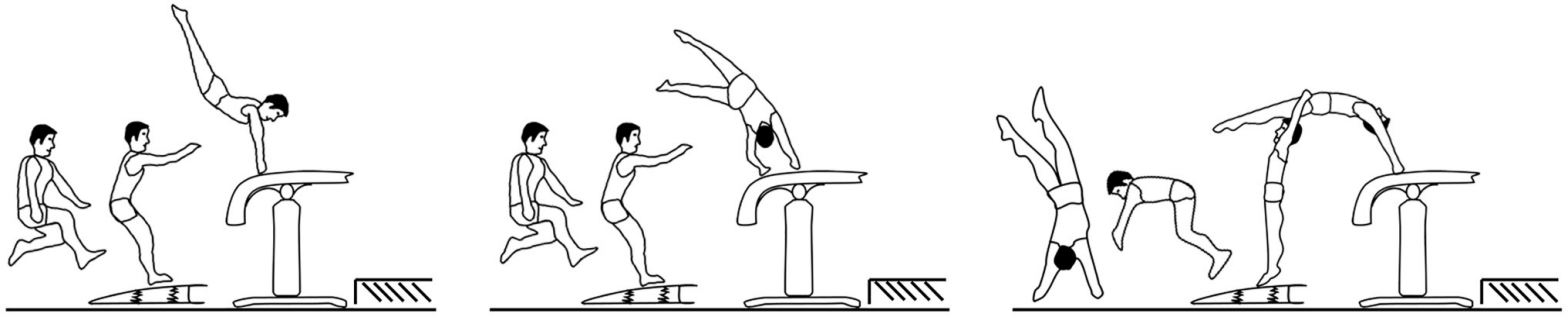
Different vault-styles. The different first flight phases of the three most important vault styles in male’s artistic gymnastics. Left: Handspring; middle: Tsukahara; right: Yurchenko [7].

Difficult vaults are assigned to a high difficulty score (D-score) in the official competition rules [3]. The vault D-score is determined, among other factors, by the number of turns around transversal and longitudinal axis during the second flight phase [4]. Since run-up speed is the most important phase of energy production [5], a high run-up speed is crucial to perform difficult vaults in competitions [4–6]. In this context it must be mentioned that the different vault styles Handspring, Tsukahara and Yurchenko (Fig 1) require different levels of run-up speed. Handspring and Tsukahara (Ha/Ts) style vaults are generally performed with higher run-up speeds than Yurchenko (Yu) style vaults [7]. Contrary to Ha/Ts vaults (forward take-off from vaulting board), for Yu vaults, the gymnasts perform a round-off in front of the vaulting board and backwards handspring on the vaulting table. On the one hand, these elements allow the athlete to generate the necessary angular momentum and vertical velocity for the second flight phase, but on the other hand they also restrain the run-up speed due to the limiting mechanical behavior of locomotion of the upper limbs [8]. Nevertheless, in order to perform a more difficult vault and thus to have a better chance to achieve a high final score, in general a greater run-up speed is required [7]. The vault run-up was previously described as a 20m-acceleration followed by 5m of maintaining the speed in order to hit the vault board optimally and to be able to perform the planned vault [2]. Further, Veličković, Petković [9] found that top gymnasts have different velocity patterns during the vault run-up than less skilled gymnasts. Nevertheless, the vault run-up seems to be a highly standardized target-directed sprint [10], due to the limited run-up distance and the performance of a complex skill at the end of the run-up. However, to this day it is unclear to what extent step kinematics during the run-up are important to attain a high run-up speed and to perform a difficult vault. Further, the contribution of physical parameters, such as sprint speed, explosive strength (muscular power, rate of force development) and reactive strength (performance in a short (< 200 ms) stretch-shortening cycle) to a high run-up speed remain elusive. In general for linear short sprints in other sports (e.g. athletics, football), step kinematics and explosive and reactive strength are considered to be important in order to achieve a high sprint speed [11–13]. In artistic gymnastics, the importance of lower body power (sprint speed, explosive strength, reactive strength) for the performance on vault is generally recognised as essential [14] but remains mostly unexplored. In this context, Tashiro, Takata [15] showed that 25m sprint speed, run-up speed and scores on vault are significantly correlated. Further, Koperski, Kochanowicz [16] found that a take-off from the spring board is similar to a drop jump and additionally Bradshaw and Le Rossignol [17] showed that an effective use of the strech-shortening-cycle during take-off from the vaulting board results in a better vaulting performance of 8 to 15 years old girls.

A complete overview of the physical parameters that determine run-up speed and therefore, the potential to perform difficult vaults, would help coaches to compile even more specific training programs for their athletes in order to succeed. Moreover, it is important to observe the development of the physical parameters, in order to optimize the training programs and the individual progress of each gymnast during his carreer.

Hence, the first aim of our study was to explore the physical determinants of vault performance and for this purpose to investigate how run-up kinematics on vault (maximal speed, step length and frequency, floor contact time) interrelate with the D-score and how maximal run-up speed on vault is correlated with lower body power (maximal 25m-sprint speed, 25m-sprint kinematics, explosive and reactive strength). The second purpose was to investigate age-related differences in physical determinants of vault performance across junior and elite top-level gymnasts. The analyses were done separately for the vault styles Ha/Ts and Yu as it might be assumed that the performance depends on different factors for these two categories of vaults [7].

## Material and methods

### Subjects

**Fig 2:**
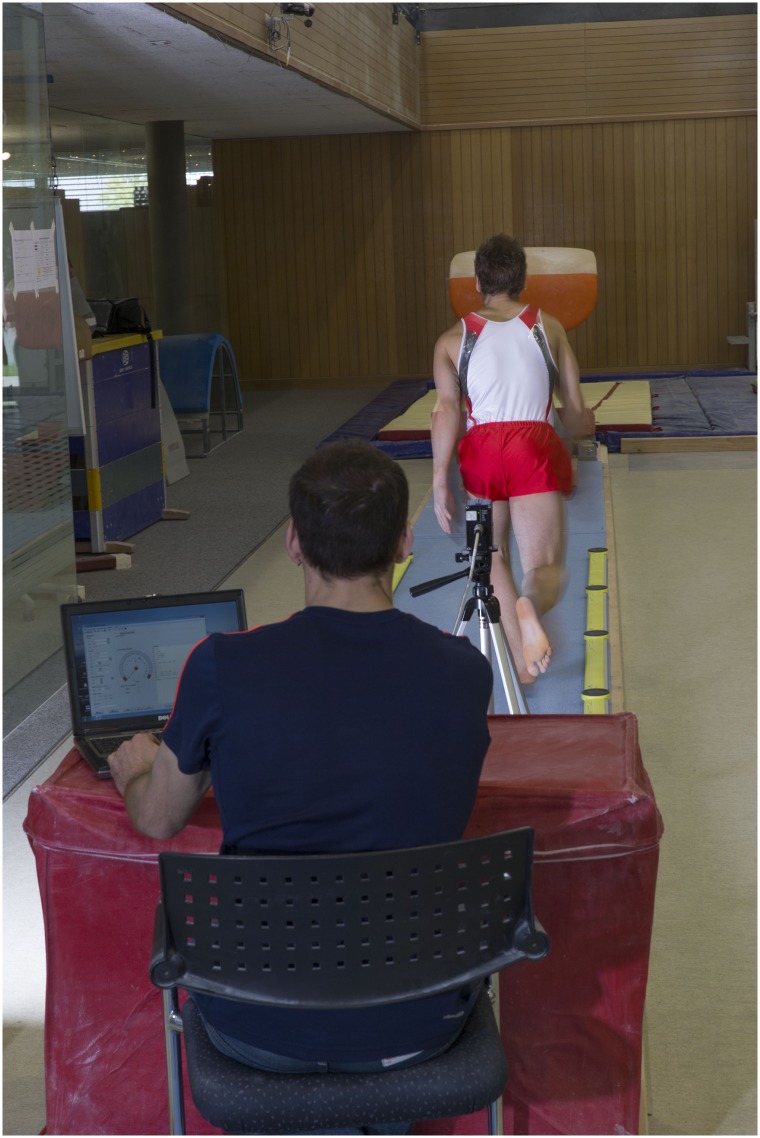
Experimental set-up. Measurement of maximal run-up speed (laser: LDM301a, Jenoptik, Rostock, Germany) and step kinematics (step frequency, step length, ground contact time) on vault (Opto Jump, Microgate, Italy). The individual in this figure has given written informed consent (as outlined in PLOS consent form) to publish these case details.

The data of 47 gymnasts of the male junior and elite national team (19.18 ± 3.42 years; age range: 14.3 to 28.3 years; height: 166.69 ± 5.51 cm; weight: 62.23 ± 7.56 kg) were included to the analysis. The Ethics Committee Bern approved the study (Project-ID: 2018–00742) and permitted the publication of the anonymized data for “further use without consent" according to paragraph 32 of the declaration of Helsinki and to Art. 34 of the Human Research Ordinance (HRO) of the Swiss Confederation. Gymnasts were not recruited specifically, but the performance testing procedures were an annual recurring part of the usual preparation of all members of the Swiss national gymnastics teams. Athletes were informed about the testing procedures before the different tests. The study was conducted in accordance with the current version of the Declaration of Helsinki, the ICH-GCP, ISO EN 14155, and all national legal and regulatory requirements. The individual in this manuscript has given written informed consent (as outlined in PLOS consent form) to publish these case details (Fig 2).

### Procedures

For the purpose of performance testing, maximal run-up speed, run-up kinematics and D-score on vault as well as lower body power (maximal 25m-sprint speed, 25m-sprint kinematics, explosive- and reactive strength) of the members of the male junior and elite national team were assessed during three consecutive years. The testing procedures were performed in accordance to the ‘Manual of Performance Testing for Swiss Olympic Medical Centers’ [18] in an early preparatory phase, in order to provide training recommendations for athletes and coaches. Within two consecutive days at the beginning of the week and after an individual warm-up, the athletes performed two competition vaults under training conditions (soft landing mat allowed), two maximal linear 25m-sprints as well as an explosive and reactive strength test. During vault run-up and 25m-sprint, maximal speed and step kinematics were assessed. Maximal speed (v_peak_vault; v_peak_sprint) was measured using a laser measurement system (LDM301a, Jenoptik, Rostock, Germany) placed 30 m away from the vaulting table (height: 1m). For the calculation of maximum speed, the raw (100-Hz) laser position data were clustered into and averaged within consecutive 0.04-s bins, thus yielding a 25-Hz position-time signal. Step kinematics of vault run-ups and 25m-sprints were assessed with Opto Jump Next (Microgate, I) with a measuring frequency of 1000 Hz (Fig 2). Mean step frequency (SF_vault; SF_sprint), mean step length (SL_vault; SL_sprint) and mean ground contact time (t_cont_vault; t_cont_sprint) of the last four steps of the vault run-up and 25m sprint were included in the evaluations. Moreover, the D-score of the performed vaults were recorded. From these measurements only the vault with the better execution (judged by an expert) and the faster 25m-sprint were included for the analyses. If a gymnast was able to perform vaults from both vault styles (Ha/Ts and Yu) only the vault with the highest D-score and best execution was chosen for the evaluations. If a gymnast participated more than once at the performance testing, only the results of the test with the highest D-score was used for the calculations of the study. Explosive strength was determined executing three Countermovement jumps (CMJ), three Squat jumps (SJ) and three single leg Countermovement jumps (mean between left and right: SL-CMJ) and reactive strength was measured from two drop jumps (DJ) from a 20, 40 and 60 cm step onto a force plate (MLD Test Evo 2, SPSport, Innsbruck, Austria). The maximal value of the three CMJ, SJ and SL-SJ (relative peak power: Pmax_rel; W/kg) and the maximal value of DJ’s (reactive index: jump height / ground contact time; cm/10∙s) were included in the calculations.

### Statistical analysis

Descriptive statistics were performed summarized per vault style (Ha/Ts; Yu) and age-group (under (U) 17; U19; U21; >21 years). In order to calculate the physical determinants of D-score, the data was separated into Ha/Ts (n = 33) and Yu style vaults (n = 14). The relationships between the measured parameters were calculated using Spearman’s Rho (r) and explained variance (R^2^) and displayed in a deterministic model of D-score. Correlation coefficients were compared according to Eid, Gollwitzer [19]. The model was created based on findings of previous studies that found 1) that D-score on vault is closely related with run-up velocity [7], 2) that maximal sprint speed represents the exploitable potential during run-up, 3) that (any) sprint speed is determined as the product of step length and step frequency [13] and 4) that lower body power is important in order to perform fast sprints over short distances [20]. Age-related differences of D-score, run-up speed, run-up kinematics and lower body power were calculated only for Ha/Ts vaults, due to the small number of athletes that performed Yu vaults. Kruskal Wallis test was used to calculate the differences across all age-groups and Mann-Whitney-U-Tests (post hoc) and effect sizes were calculated ($r=Z/\sqrt{N}$) [21] (rated according to Cohen [22]: small: ≥ 0.1; medium: ≥ 0.3; large: ≥ 0.5) to assess differences between consecutive age-groups. The level of statistical significance was set to p < 0.05. P-values were adjusted using the Holm-Bonferroni correction [23]. All calculations were performed using SPSS 22 software (SPSS, Inc., Chicago, IL).

## Results

Due to measurement errors, the step frequency of three vault run-ups (Ha/Ts) and two 25m-sprints as well as the step length of one run-up were excluded before the evaluations.

### Physical determinants of vault performance

**Fig 3:**
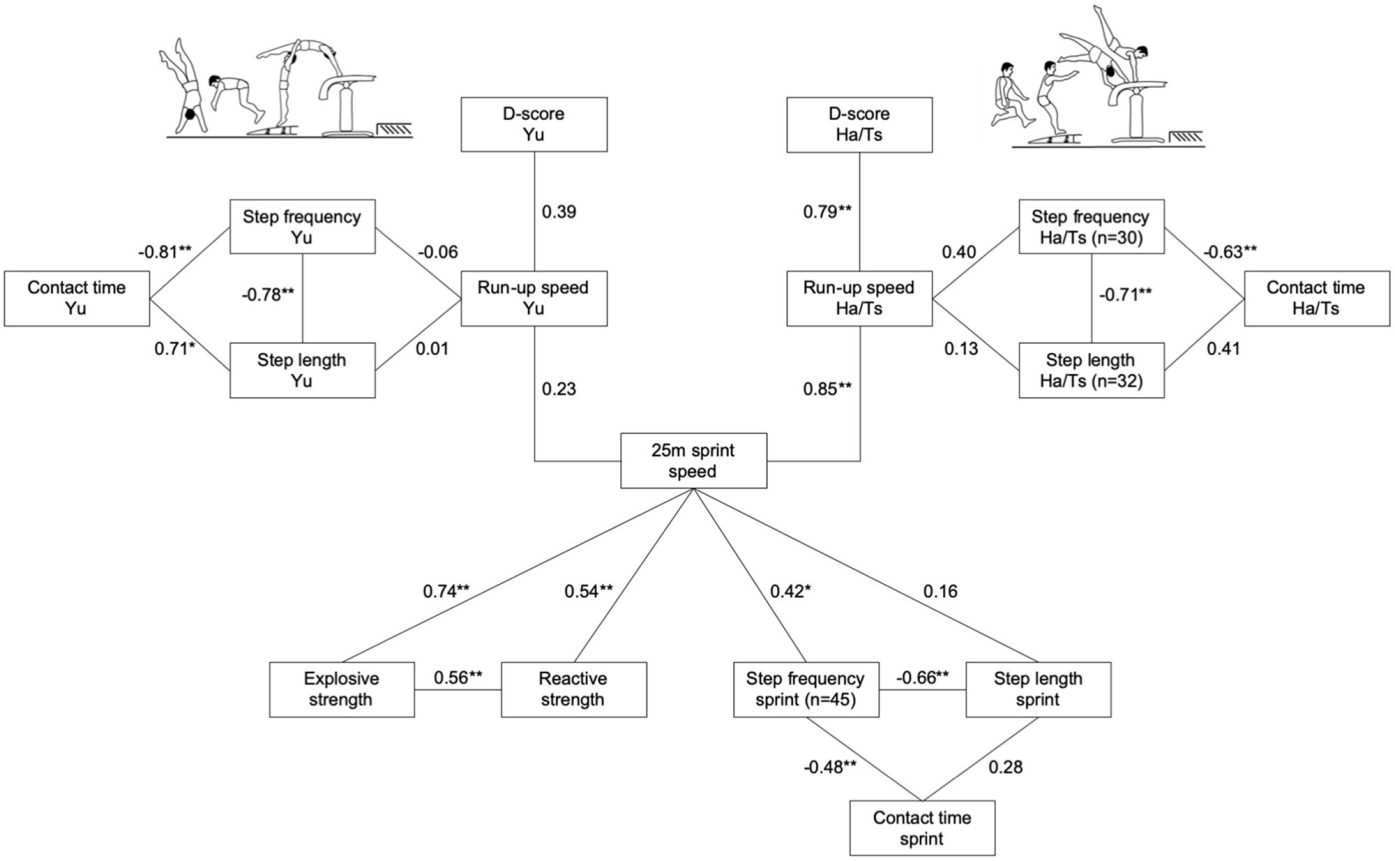
Deterministic model of vault performance. Deterministic model (relationships = Spearman’s Rho) of the D-score of male’s Handspring/Tsukahara- (Ha/Ts) and Yurchenko-style vaults (Yu) (*: p < 0.05; **: p < 0.01) (Yu: n = 14; Ha/Ts: n = 33; 25m-sprint kinematics, explosive and reactive strength: n = 47).

The run-up speed of Ha/Ts and Yu explained 62% and 15% of the variance of D-score (R^2^), respectively. The variation of run-up speed was explained from 5% (Yu) to 72% (Ha/Ts) by the 25m sprint speed. Further, explosive strength, reactive strength and step frequency explained 55%, 29% and 17% of the variation of sprint speed (Fig 3). Step frequency of Ha/Ts and 25m-sprint correlated slightly better (non-significant: p = 0.15) with run-up speed and 25m sprint speed than step length.

There were no significant differences of lower body power (maximal 25m-sprint speed, 25m-sprint kinematics, explosive- and reactive strength) between gymnasts performing Ha/Ts and Yu, but significant differences between the run-up kinematics of these two different vault styles (Table 1).

**Table 1:** Differences between athletes performing Handspring/Tsukahara and Yurchenko.

	Ha/Ts (n = 33)	Yu (n = 14)
**D-score** [points]	4.67 *± 0*.*51*	4.86 *± 0*.*52*
**v_peak_vault** [m/s]	8.01 *± 0*.39***^L^	7.49 *± 0*.*18*
SF_vault [Hz]	4.63 *± 1*.02**^M^	4.17 *± 0*.*42*
SL_vault [m]	1.64 *± 0*.27*^M^	1.83 *± 0*.*21*
t_cont_vault [s]	0.12 *± 0*.01*^M^	0.13 *± 0*.*03*
**v_peak_sprint** [m/s]	8.19 *± 0*.*39*	8.22 *± 0*.*39*
SF_sprint [Hz]	4.41 *± 0*.*76*	4.50 *± 0*.*22*
SL_sprint [m]	1.73 *± 0*.*10*	1.77 *± 0*.*08*
t_cont_sprint [s]	0.12 *± 0*.*01*	0.12 *± 0*.*01*
**CMJ** (Pmax_rel) [W/kg]	60.85 *± 8*.*02*	61.44 *± 6*.*27*
**SJ** (Pmax_rel) [W/kg]	55.33 *± 6*.*13*	56.29 *± 5*.*25*
**SL-CMJ** (Pmax_rel) [W/kg]	37.14 *± 4*.*16*	37.26 *± 4*.*83*
**DJ** [cm/10∙s]	22.75 *± 4*.*08*	24.65 *± 3*.*15*
**Body weight** [kg]	62.47 *± 7*.*89*	61.66 *± 6*.*98*
**Height** [m]	1.67 *± 0*.*06*	1.67 *± 0*.*05*
**Age** [y]	19.10 *± 3*.*59*	19.36 *± 3*.*09*

Mean values (± standard deviation) and significant differences (Mann-Whitney-U-Test) between athletes performing Handspring/Tsukahara (Ha/Ts) and Yurchenko (Yu) vaults of difficulty value (D-score), vault run-up and 25m-sprint kinematics (v_peak: maximal speed, SL: mean step length of the last four steps; SF: step frequency of the last four steps; t_cont: mean ground contact time of the last four steps) explosive strength (Countermovement- (CMJ), Squat- (SJ) and single leg Countermovement Jump: SL-CMJ), reactive strength (DJ) as well as anthropometric parameters (*: p < 0.05; **: p < 0.01; ***: p < 0.001; ^M^: medium effect size: r > 0.3; ^L^: large effect size: r > 0.5 in relation to Athletes performing Yurchenko vaults).

### Age-related differences across junior and elite level gymnasts

Kruskal Wallis test showed significant differences across the different age categories for all measured parameters (p < 0.05) except for step length, step frequency and contact time of vault run-up and 25m sprint (range of p-value: 0.08 to 0.23).

Post-hoc analysis (Mann-Whitney-U) revealed a significant increase of the D-score (+11.6%; p < 0.01), when considering the overall development (U17 vs. >21). Between consecutive age-groups the D-score rose in mean 4% (p > 0.05).

In general, the best performance for most of the physical parameters was measured for the >21 age-group. The run-up speed was significantly higher with increasing age (U17 vs. U19; U19 vs. U21). Anthropometric parameters (body weight and height) were only significantly different between the U17 and U19 age-groups and lower body power (sprint speed, explosive strength) was only significantly higher in the U21 compared to U19 (Table 2).

**Table 2:** Age-related differences.

	U17 (n = 8)	U19 (n = 11)	U21 (n = 7)	>21 (n = 7)
**D-score** [points]	4.40 *± 0*.*43*	4.51 *± 0*.*44*	4.86 *± 0*.*67*	5.03 *± 0*.*21*
**v_peak_vault** [m/s]	7.60 *± 0*.*38*	7.94 *± 0*.28*^M^	8.27 *± 0*.21*^L^	8.31 *± 0*.*16*
SF_vault [Hz]	4.64 *± 0*.*42*	4.63 *± 0*.*26*	4.81 *± 0*.*46*	4.93 *± 0*.*29*
SL_vault [m]	1.63 *± 0*.*11*	1.71 *± 0*.*10*^*M*^	1.71 *± 0*.*11*	1.69 *± 0*.*10*
t_cont_vault [s]	0.12 *± 0*.*01*	0.12 *± 0*.*01*	0.12 *± 0*.*01*^*M*^	0.11 *± 0*.*01*^*M*^
**v_peak_sprint** [m/s]	7.94 *± 0*.*34*	8.10 *± 0*.*25*	8.31 *± 0*.28*^L^	8.47 *± 0*.*12*^M^
SF_sprint [Hz]	4.54 *± 0*.*24*	4.50 *± 0*.*23*	4.67 *± 0*.*32*	4.68 *± 0*.*26*
SL_sprint [m]	1.67 *± 0*.*08*	1.76 *± 0*.12*^L^	1.74 *± 0*.*06*	1.75 *± 0*.*09*
t_cont_sprint [s]	0.12 *± 0*.*01*	0.12 *± 0*.*01*	0.12 *± 0*.*01*^*M*^	0.11 *± 0*.*01*^*M*^
**CMJ** (Pmax_rel) [W/kg]	55.43 *± 7*.*82*	58.73 *± 6*.*73*^M^	65.31 *± 6*.39*^M^	65.93 *± 7*.*38*
**SJ** (Pmax_rel) [W/kg]	51.26 *± 6*.*53*	53.70 *± 5*.*63*	59.75 *± 4*.*84*^M^	58.14 *± 3*.*95*
**SL-CMJ** (Pmax_rel) [W/kg]	33.77 *± 3*.*58*	36.19 *± 3*.*04*^M^	39.91 *± 3*.75*^M^	39.72 *± 3*.*78*
**DJ** [cm/10∙s]	19.03 *± 3*.*90*	22.11 *± 3*.*62*^M^	24.46 *± 2*.*24*^M^	26.31 *± 2*.*63*^M^
**Body weight** [kg]	51.69 *± 6*.*39*	64.81 *± 4*.37***^L^	67.17 *± 6*.*11*	66.41 *± 2*.*50*
**Height** [m]	1.60 *± 0*.*05*	1.69 *± 0*.04**^L^	1.68 *± 0*.*05*	1.69 *± 0*.*04*
**Age** [y]	16.39 *± 0*.*28*	18.35 *± 0*.58***^L^	20.46 *± 0*.93***^L^	24.76 *± 1*.93***^L^

Mean values (± standard deviation) and significant differences (Mann-Whitney-U-Test) between age-groups of difficulty value of Handspring / Tsukahara vaults (D-score), vault run-up and 25m-sprint kinematics (v_peak: maximal speed, SL: mean step length of the last four steps; SF: step frequency of the last four steps; t_cont: mean ground contact time of the last four steps) explosive strength (Countermovement- (CMJ), Squat- (SJ) and single leg Countermovement Jump: SL-CMJ), reactive strength (DJ) as well as anthropometric parameters. (*: p < 0.05; **: p < 0.01; ***: p < 0.001; ^M^: medium effect size: r > 0.3; ^L^: large effect size: r > 0.5 in relation to previous age group).

## Discussion

This is the first study to calculate the physical determinants of D-score on vault separately for Ha/Ts and Yu-style vaults. Secondly, age-related differences of D-score, maximal run-up speed, run-up kinematics and lower body power (25m-sprint kinematics, explosive and reactive strength) were analyzed across male junior and elite top-level gymnasts performing Ha/Ts-style vaults.

The cross-sectional data in this study that was gathered over a period of three years, may influence the results. Therefore, interpretations for the longitudinal development of athletes’ performance should be made carefully. However, our data show the actual performance level of different age-groups of a large cohort of top-level gymnasts. Therefore, the level of the different physical parameters at the different age-groups can be used as reference values to verify the longitudinal development of each athlete.

### Physical determinants of vault performance

The data revealed strong relationships between D-score of Ha/Ts vaults and run-up speed. Therefore, the importance of a high run-up speed to perform difficult Ha/Ts vaults could be confirmed. Further, the run-up speed of Ha/Ts was correlated significantly with 25m-sprint speed. Therefore, our results are in line with Tashiro, Takata [15] that also reported strong relationships between these three parameters (r > 0.72). Hence, it may be assumed that general sprinting ability represents the exploitable speed potential for the vault run-up and therefore for the performance on vault. In addition, this corresponds with the findings of Gross, Buchler Greeley [24] that found similar relationships between the performance (jump height), approach speed and sprint performance of female pole vault athletes. These similarities may be surprising, but both, pole vaulting and the gymnastics vault are technically very demanding disciplines, where athletes have to hit a target at the end of a submaximal sprint and where the approach speed determines to a large extent the performance. However, an important difference between these two disciplines is the limited run-up distance (25m) of the gymnastics vault. Within the run-up distance gymnasts rely on a fixed run-up pattern in order to hit the vault board optimally [25]. Consequently, a higher step frequency may be the only possibility to increase the run-up velocity when performing more difficult vaults. This may be confirmed with the slightly higher (but not significantly different) correlation coefficient between step frequency and run-up speed compared to the relationship between step length and run-up speed. Interestingly, the step frequency of the 25m-sprint in this study was similarly related to the 25m-speed. Therefore, gymnasts seem to use a similar sprint pattern to achieve a high 25m-sprint speed as if running-up to perform a vault. These findings are opposite to the results of Tashiro, Takata [15] that observed different running techniques during the run-up on vault and a 25m-sprint of elite gymnasts. In addition, our results are contrary to general findings concerning the kinematics of linear short sprints. In this context, sprint speed is determined as the product of step length and step frequency [13], but usually, step length is considered—in particular during acceleration—to be more important for better sprint performances than step frequency [26, 27]. As a result, coaches should compile training programs particularly to improve the step frequency in order to increase the run-up speed on vault.

Further, our results showed significant correlations between 25m-sprint speed and explosive- as well as reactive strength. Sprint speed strongly depends on the horizontal force impact generated during the short ground contact time. Hence, peak power of the lower limbs can be considered a determining factor of high speeds during short linear sprints [11]. Thus, our results are in line with other studies that found strong correlations between maximum sprint speed over a short distance and relative power output performing Countermovement Jumps, Squat Jumps and Drop Jumps [26, 28]. Hence, explosive and reactive strength, may be important (indirect) determinants of run-up velocity of Ha/Ts vaults. Therefore, coaches should be aware that the optimization of these parameters may enhance the potential to perform more difficult Ha/Ts vaults.

In contrast to Ha/Ts, we found no significant relationships between the D-score and run-up kinematics neither between run-up speed and 25m-sprint speed of Yu vaults. Hence, limiting factors of performing a difficult Yu vault are probably rather high technical and mental requirements of the round-off in front of the springboard and the backwards handspring on the vaulting table [7] than physical parameters. Hence, run-up speed and step pattern may be adapted to the mental and technical level of the gymnast. Nevertheless, lower body power (25m-sprint speed and kinematics, explosive and reactive strength) of gymnasts performing Yu vaults was not significantly different from gymnasts performing Ha/Ts. This suggests that the choice to perform a Yu vault is not primarily made because a gymnast has less lower body power but rather in consideration of the technical and / or mental abilities of the gymnast. Furthermore, lower body power is not only important to perform difficult vaults. It is even considered a general basic precondition in gymnastics and in particular, for the successful performance on floor [29, 30].

At this point it must be said that the results of Yu may be somewhat influenced by the small number of gymnasts that participated in this study. Nonetheless, all the athletes were members of the national team and therefore the results represent the current status of international and national top-level gymnasts.

### Age-related differences across junior and elite level gymnasts

Our results show a significant increase of D-score (> 0.6 points) over all age-groups (from U17 to >21) and a continuous (but non-significant) increase between all consecutive age-groups across junior and elite level. In order to illustrate this, it can be said that compared to the U17, 50% of the >21 gymnasts performed a 180° and 50% a 360° turn more around the longitudinal axis within the somersault during the second flight phase, since an additional half turn is generally rewarded with +0.4 points in the CoP [3]. This indicates that already younger gymnasts are able to perform quite difficult vaults and that improving the D-score on vault occurs slowly but steadily. Since the performance of the vault is equally dependent of the execution, it may be assumed that gymnasts invest a lot of time to improve the execution of a vault before they try to perform a more difficult vault.

The comparison between the U17 and U19 age-group showed significantly higher maximal run-up speed, step length of 25m-sprint, height and weight for the U19. It can be assumed that training, growth and maturation are major influencing factors that have led to the significant differences between these two age-groups. Increased height and consequently longer limbs of the gymnasts result in a larger step length, which in turn leads to an improvement of sprint speed [13, 31]. However, for gymnasts, changes in step length during vault run-up entails an adaptation of their usual standardized run-up pattern. Therefore, gymnasts may become unsettled performing their vault as they have to relearn hitting the vault board with the new step pattern. Since the step length remains similar across the subsequent age categories (U21, >21), it might be assumed that gymnasts always retain the same step length after growth is completed. Therefore, the relearning and subsequent stabilization of the new run-up pattern during and after maturation seems to be crucial. In this case the only possibility to increase run-up speed is, as previously mentioned, to run-up with a higher step frequency.

The U21 age-group showed significantly higher run-up speeds, 25m-sprint speeds and explosive strength than the U19 but similar height and weight. Previous studies have shown, that improvements of explosive strength are important to increase sprint speed over short distances [32]. Therefore, it is likely that the higher explosive strength of the U21 age-group enables these gymnasts to achieve a higher 25m-sprint and consequently to increase the potential to run-up with a higher speed performing Ha/Ts vaults.

Comparing the U21 and >21 age-groups, no significant differences could be found for any of the measured parameters. With regard to the similar run-up speed, this may indicate that a certain run-up speed is needed to achieve a certain D-score and that most gymnasts achieve this speed around the age of 21. Further, comparing the difference between run-up speed and 25m-sprint speed in the U21 age group and the >21, it is apparent that run-up speed for Ha/Ts of the U21 is only slightly lower than maximal 25m-sprint speed but there is a larger difference between run-up speed and 25m-sprint speed in the >21 age-group. Consequently, the younger athletes have to exploit almost their entire speed potential for their performance on vault. Running-up with an almost maximal speed may interfere with the execution of the vault and mistakes may happen more often. Therefore, the slightly higher (medium effect) 25m-sprint speed of the >21 age-group may enable the older gymnasts to run-up with a submaximal speed and this could contribute to a more consistent vault performance.

Finally, the >21 age-group achieved in general the highest values in most of the measured parameters. Therefore, it can be assumed that the major changes of lower body power and run-up speed occur up to the age of 21. This is contrary to the findings of [33]. They observed a linear improvement of the run-up speed from 12 to 18 years of 0.2 m/s per year, but a stagnation from the age of 18.

**Fig 4:**
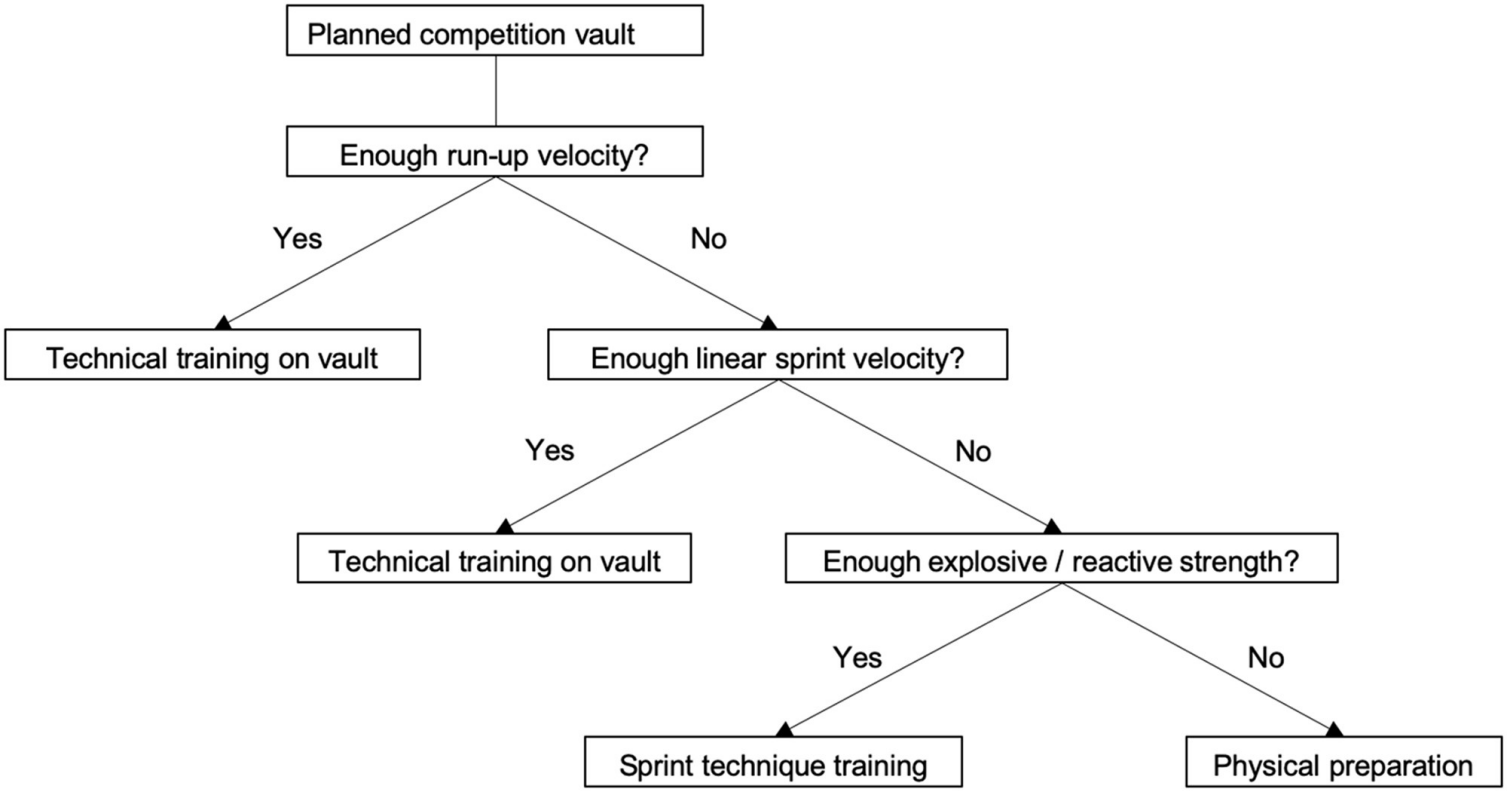
Flow-chart for training recommendations. Decision tree to derive training recommendations from a physical test battery of the lower body in consideration of the performance on vault.

Our results show the importance of run-up kinematics and lower body power for the vault performance. However, when training a new vault, coaches and athletes mainly focus on the enhancement of technical skills and often do not consider adequately the level of physical parameters that are required to reach sufficient run-up speed. In order to optimize the development of the vault performance and the individual physical training, it is important that physical parameters are regularly assessed in relation with the vault performance. From the performance testing, results detailed training recommendations should be derived on the basis of the decision tree displayed in Fig 4.

## Conclusions

The results of this study revealed that the performance (D-score) of Ha/Ts (but not Yu vaults) strongly depends on a high run-up speed. In line with this, the run-up speed of Ha/Ts is closely related to 25m-sprint speed. Furthermore, to the ability to create a high step frequency may be an important factor to create high speeds within the limited run-up distance on vault. In addition, explosive and reactive strength are strongly correlated with the 25m-sprint speed. Consequently, an optimization of these determining physical parameters may contribute to a better vault performance.

When regarding the age-related differences of athletes performing Ha/Ts vaults, we found a slow but steady increase of D-score across all age-groups of junior and elite level. The high technical requirements of more difficult vaults and the concentration on improving the execution of already mastered vaults may be the reasons for the slow but steady progresses.

The major changes of the vault run-up speed occur up to the age of 21. In this context, we assumed that the significant differences of run-up speed between the U17 and U19 age-groups are mainly encompassed by the effects of growth and maturation and therefore by a greater step length during run-up. Whereas, the significant differences of run-up speed between the U19 and U21 age-groups may be attributed to the higher level of lower body power (25m-sprint speed and explosive strength) of the older gymnasts.

The gymnasts of the >21 age-group achieved the best performances of all age-groups in most of the measured parameters. The higher run-up speed and better lower body power of gymnasts may contribute to a more consistent vault performance.

Finally, a regular assessment of vault run-up kinematics and lower body power of elite gymnasts may enable coaches to observe the development of the athletes. Furthermore, the derivation of training recommendations from regular performance testing may contribute to the optimization and individualization of the training process.

## Supporting information

S1 Table Raw data. Complete data of the present study.
(PDF)
