# Supplementary material for: Physical determinants of vault performance and their age-related differences across male junior and elite top-level gymnasts
Source: PLoS One. 2019 Dec 5;14(12):e0225975. doi: 10.1371/journal.pone.0225975 (PMC6894803; doi:10.1371/journal.pone.0225975)
Supplement: S1 Table — Complete data of the present study. (PDF) [file pone.0225975.s001.pdf]

Athlete Nr.	Age Group	Vault Group	D-score [points]	v_peak_vault [m/s]	SF_vault [Hz]	SL_vault [m]	t_cont_vault [s]	v_peak_sprin t [m/s]	SF_sprint [Hz]	SL_sprint [m]	t_cont_sprint [s]	CMJ (Pmax_rel) [W/kg]	SJ (Pmax_rel) [W/kg]	SL-CMJ (Pmax_rel) [W/kg]	DJ [cm/10·s]	Body weight [kg]	Height [m]	Age [y]
1	U17	Ha/Ts	4.8	7.66			0.12	7.80	4.38	173.75	0.11	47.92	47.15	32.82	23.40	1.55	42.50	15.30
2	U17	Ha/Ts	4.0	7.76	4.27	177.75	0.14	7.91	4.17	177.75	0.14	53.25	46.75	30.81	17.60	1.65	57.50	15.40
3	U17	Ha/Ts	4.8	8.09	5.31	154.00	0.11	8.14	4.92	162.50	0.12	58.54	49.39	37.03	18.20	1.60	49.60	15.10
4	U17	Ha/Ts	4.0	7.06	4.15	176.00	0.13	8.33	4.56	168.00	0.13	53.77	51.37	31.92	17.10	1.63	56.70	15.40
5	U17	Ha/Ts	4.8	8.12	5.09	151.25	0.11	8.14	4.84	156.00	0.12	70.03	64.87	37.85	25.50	1.61	57.90	15.40
6	U17	Ha/Ts	4.8	7.50	4.45	171.50	0.13	8.24	4.45	171.50	0.12	62.51	56.94	38.91	19.60	1.66	55.60	15.60
7	U17	Ha/Ts	4.0	7.40	4.64	158.25	0.11	7.68	4.53	166.25	0.12	47.81	47.10	29.99	12.90	1.60	51.20	15.00
8	U17	Ha/Ts	4.0	7.20	4.59	154.25	0.13	7.30	4.49	158.25	0.14	49.63	46.50	30.83	17.90	1.50	42.50	15.90
9	U19	Ha/Ts	4.8	8.11	4.60	176.75	0.13	8.27	4.42	180.50	0.14	60.13	56.53	32.52	21.90	1.73	71.00	16.60
10	U19	Ha/Ts	4.8	8.39	4.69	178.00	0.12	8.54	4.27	188.75	0.10	64.80	58.84	40.21	18.80	1.71	61.30	18.00
11	U19	Ha/Ts	4.4	7.67		188.00	0.14	7.73		195.75	0.14	49.70	47.28	30.96	18.20	1.67	65.60	17.50
12	U19	Ha/Ts	4.8	8.17	4.56	177.75	0.11	8.12	4.68	174.25	0.11	56.27	52.73	35.37	24.60	1.64	62.40	17.20
13	U19	Ha/Ts	4.0	7.79	4.81	155.00	0.12	8.05	4.97	149.00	0.11	62.84	61.50	35.87	24.70	1.62	67.30	18.30
14	U19	Ha/Ts	5.2	8.19		165.50	0.12	8.19		175.50	0.11	61.47	55.10	37.34	25.70	1.72	72.00	17.90
15	U19	Ha/Ts	4.8	7.90	4.46	171.25	0.11	7.97	4.45	177.75	0.12	55.30	49.20	35.60	20.70	1.72	60.60	16.80
16	U19	Ha/Ts	4.0	8.04	4.45	174.50	0.13	8.33	4.43	178.75	0.12	73.51	62.69	39.39	27.00	1.71	68.80	16.90
17	U19	Ha/Ts	4.8	7.93	5.16	155.25	0.13	8.24	4.69	170.75	0.13	55.22	46.63	40.67	24.80	1.65	59.70	17.80
18	U19	Ha/Ts	4.0	7.66	4.23	179.25	0.13	7.94	4.35	172.25	0.13	52.40	49.58	34.77	21.40	1.70	63.00	17.00
19	U19	Ha/Ts	4.0	7.44	4.72	160.50	0.13	7.73	4.28	175.25	0.13	54.35	50.66	35.39	15.40	1.71	61.20	16.80
20	U21	Ha/Ts	4.8	8.22	4.12	188.00	0.12	8.34	4.22	186.50	0.12	68.06	65.52	39.87	23.60	1.70	62.30	21.00
21	U21	Ha/Ts	4.0	8.13	4.75	172.00	0.12	8.29	4.76	169.75	0.12	64.70	60.75	34.60	21.10	1.70	65.20	20.80
22	U21	Ha/Ts	5.6	8.34	5.37	156.00	0.11	8.46	4.94	170.75	0.11	74.40	66.87	41.10	26.10	1.65	66.10	21.30
23	U21	Ha/Ts	5.6	8.58	5.34	161.25	0.11	8.66	5.00	170.25	0.11	62.98	57.40	42.23	27.90	1.61	62.20	20.20
24	U21	Ha/Ts	4.0	7.91	4.41	180.25	0.12	7.74	4.23	179.00	0.12	53.54	55.17	36.11	25.00	1.66	62.90	18.50
25	U21	Ha/Ts	5.2	8.39	4.91	171.25	0.12	8.34	4.78	170.00	0.12	68.56	54.74	45.81	24.80	1.71	74.20	20.70
26	U21	Ha/Ts	4.8	8.35	4.80	173.75	0.13	8.37	4.75	174.00	0.12	64.90	57.81	39.65	22.70	1.75	77.30	20.70
27	>21	Ha/Ts	5.2	8.43	4.81	175.75	0.11	8.48	4.62	182.25	0.12	60.02	57.33	39.50	26.50	1.70	65.80	23.20
28	>21	Ha/Ts	4.8	8.17	4.58	176.25	0.12	8.40	4.41	179.25	0.12	77.61	62.73	38.36	29.00	1.68	63.80	25.20
29	>21	Ha/Ts	4.8	8.03	5.00	155.00	0.12	8.28	4.79	168.25	0.11	68.97	56.48	38.91	24.20	1.67	65.40	23.10
30	>21	Ha/Ts	4.8	8.28	4.57	180.50	0.12	8.39	4.28	189.50	0.12	54.03	51.82	32.79	24.00	1.75	67.10	25.90
31	>21	Ha/Ts	5.2	8.46	4.96	172.25	0.11	8.54	4.94	168.50	0.11	67.55	57.55	41.81	24.10	1.69	69.10	28.30
32	>21	Ha/Ts	5.2	8.42	5.30	162.75	0.12	8.62	4.96	167.75	0.12	66.19	57.59	41.82	30.70	1.63	63.60	23.00
33	>21	Ha/Ts	5.2	8.41	5.26	159.75	0.10	8.58	4.81	167.50	0.10	67.16	63.47	44.85	25.70	1.68	70.10	24.60
34	U17	Yu	5.2	7.55	3.65	220.75	0.22	8.37	4.57	181.75	0.12	66.51	60.40	35.64	26.00	1.63	59.90	16.40
35	U17	Yu	4.0	7.44	4.56	155.00	0.12	7.42	4.32	160.50	0.12	52.06	48.65	29.63	18.00	1.55	42.90	14.30
36	U19	Yu	4.0	7.19	4.91	174.25	0.13	7.87	4.69	170.75	0.13	59.68	53.18	37.35	23.60	1.66	60.50	16.70
37	U19	Yu	4.8	7.55	4.06	192.75	0.13	8.16	4.32	179.25	0.13	51.79	48.53	31.59	26.60	1.73	62.00	17.80
38	U19	Yu	5.2	7.50	4.03	191.50	0.13	8.42	4.57	177.75	0.12	60.59	57.69	35.75	23.90	1.69	63.10	17.40
39	U19	Yu	4.8	7.66	4.25	187.25	0.13	8.17	4.47	180.50	0.13	58.58	56.54	33.19	26.00	1.68	60.00	17.30
40	U19	Yu	4.0	7.34	4.23	170.50	0.12	7.50	4.16	179.00	0.13	55.08	51.88	34.02	24.20	1.61	56.90	16.50
41	U21	Yu	5.2	7.77	3.98	201.00	0.12	8.41	4.33	191.50	0.13	69.31	63.56	43.95	30.50	1.64	60.70	20.90
42	U21	Yu	5.2	7.45	3.93	201.00	0.13	8.14	4.49	174.50	0.13	55.69	50.91	39.97	21.60	1.68	64.60	20.60
43	U21	Yu	5.2	7.62	4.81	153.50	0.12	8.34	4.22	186.50	0.12	68.06	65.52	39.87	23.60	1.70	62.30	21.00
44	>21	Yu	5.2	7.53	4.69	147.00	0.11	8.62	4.97	166.75	0.12	68.12	60.65	44.05	29.70	1.64	63.10	23.90
45	>21	Yu	4.8	7.10	3.58	194.50	0.15	8.51	4.48	179.50	0.12	61.05	55.13	36.52	22.60	1.74	65.20	23.90
46	>21	Yu	5.6	7.52	3.85	206.00	0.13	8.76	4.68	179.00	0.12	69.06	58.25	45.56	25.00	1.75	76.00	22.60
47	>21	Yu	4.8	7.68	3.84	179.50	0.13	8.35	4.67	175.75	0.12	64.62	57.10	34.60	23.80	1.70	66.00	21.80
